# Trends in clinical development of pediatric cancer for PD-1 and PD-L1 inhibitors: an analysis of ClinicalTrials.gov

**DOI:** 10.1136/jitc-2021-002920

**Published:** 2021-09-28

**Authors:** Yi Que, Yang Hu, Dongchun Hong, Yizhuo Zhang

**Affiliations:** 1 Department of Pediatric Oncology, State Key Laboratory of Oncology in South China, Collaborative Innovation Center for Cancer Medicine, Sun Yat-sen University Cancer Center, Guangzhou, Guangdong, China; 2 Department of Medical Melanoma and Sarcoma, State Key Laboratory of Oncology in South China, Collaborative Innovation Center for Cancer Medicine, Sun Yat-sen University Cancer Center, Guangzhou, Guangdong, China

**Keywords:** pediatrics, immunotherapy

## Abstract

Compared with cytotoxic chemotherapy, radiotherapy, and surgery, positive findings have been acquired through the approach of blocking the programmed cell death protein 1 (PD-1) pathway with antibodies that exert inhibitory effects on PD-1 or cell death protein ligand 1 (PD-L1). Results from clinical trials showed great potential in adult patients with cancers, such as melanoma, non-small cell carcinoma, and nasopharyngeal carcinoma. However, studies of checkpoint inhibitors specifically targeting PD-1/PD-L1 in pediatric patients are limited. We evaluated ongoing clinical trials using PD-1 or PD-L1 inhibitors alone or in combination with other therapies to treat pediatric cancer. The proportion of PD-1/PD-L1 combination clinical trials has increased since 2018; the three most common trials over the past 2 years used CTLA-4 monoclonal antibodies, chemotherapy, and therapies that target the vascular endothelial growth factor axis. This commentary aimed to provide trends and specific insights into methods for conducting clinical trials of immunotherapy in the pediatric population.

Immune checkpoint inhibitors, which include programmed cell death protein 1 (PD-1)/cell death protein ligand 1 (PD-L1), have substantially improved treatment outcomes and achieved a breakthrough in adult cancers in the past few years.^1–3^ The PD-1 receptor, a transmembrane glycoprotein, plays a crucial role in the immune evasion mechanism which downregulates T-cell activation. The activation of CD8^+^ T cells is suppressed by the engagement of PD-1 with PD-L1 on tumor cells. Blockade of the PD-1/PD-L1 pathway restores the ability of T cells to target tumor cells.

Currently, several immune checkpoint inhibitors—nivolumab, pembrolizumab, avelumab, and toripalimab—have been approved by the Food and Drug Administration, European Medicines Agency, and China Food and Drug Administration for use in adult cancers. There are also several PD-1 antibodies—REGN2810, AMP-224, and BGB-A317—still in phase I clinical trials. The main PD-1 and PD-L1 antibodies are summarized in online supplemental table 1.

10.1136/jitc-2021-002920.supp1 Supplementary data

Studies of checkpoint inhibitors specifically targeting PD-1/PD-L1 in pediatric patients are limited.^4^ Results from an interim analysis of the KEYNOTE-051 trial indicated that pembrolizumab had antitumor activity against relapsed or refractory Hodgkin’s lymphoma (HL) and some uncommon tumor types (mesothelioma and adrenocortical carcinoma).^5^ In addition, nivolumab was safe and displayed antitumor activity in the pediatric population with relapsed or refractory non-central nervous system (CNS) solid tumors or lymphoma.^6^ Nevertheless, when used alone, neither nivolumab nor pembrolizumab showed activity in the sporadic pediatric solid tumor histotypes. For non-HL tumors, anti-PD-1 showed modest efficacy, which might be due to the low tumor mutational burden (TMB) or low PD-L1 expression in the tumor microenvironment. Currently, there is significant evidence that combination immunotherapy shows clinical activity compared with PD-1 alone in adult cancer types. However, the status of combination therapy in pediatric cancer remains unknown. Thus, there is still great potential for researchers to explore whether PD-1 monotherapy with or without other regimens is effective in treating pediatric tumors. This commentary provides an assessment of ongoing clinical trials using PD-1 or PD-L1 inhibitors alone or in combination with other therapies and provides specific insights into the methods for conducting clinical trials of immunotherapy in the pediatric population.

We first initiated a search on ClinicalTrials.gov on September 8, 2020, using the following search terms: PD-1 OR PD-L1 OR Nivolumab OR Pembrolizumab OR Durvalumab OR Toripalimab OR Sintilimab OR Atezolizumab OR avelumab OR REGN2810 OR AMP-224 OR AMP-514 OR PDR-001 OR BCD-100 OR TSR-042 OR JNJ-63723283 OR PF-0681591 OR BI-754091 OR SHR-1210 OR JS001 OR IBI308 OR GB226 OR GLS-010 OR LZM009 OR HX008 OR BGB-A317 OR M7824 OR CX-072 OR FAZ-053 OR LY-3300054 OR CA-170 OR SHR-1316 OR KN035 OR ZKAB001 OR CS1001 OR BAT1306. Using this search strategy, 2688 trials were identified. We limited trials to ongoing clinical trials in which the status was recruiting or not yet recruiting, and the age of patients enrolled was ≤18 years. Then, 150 trials were identified to be screened. Next, we excluded 10 duplicate trials, 7 trials that did not involve PD-1 antibody therapy, 1 trial that was not related to tumor therapy, 22 observational trials, and 12 trials that did not meet the inclusion age requirements. After examination for manual categorization, 98 trials were selected for review (online supplemental table 2).

Judging from the analysis, 24 of the 98 interventional trials were related to PD-1/PD-L1 monoclonal antibodies alone, and 74 were related to PD-1/PD-L1 antibodies alongside other treatments. We found that the percentages of PD-1/PD-L1 monotherapy clinical trials and combination clinical trials were 32% and 68%, respectively, before 2018. However, the ratio decreased to 21.9% vs 78.1% in the ensuring 2 years. Phase II and phase I/II accounted for 23.5% and 44.9%, respectively, indicating that the application of PD-1 antibody in pediatric patients with tumors is still in its infancy and trial stage. Pembrolizumab, nivolumab, and atezolizumab are drugs commonly used as monotherapies (online supplemental figure 1). Sintilimab (NCT04400851 and NCT04412408) and ZKAB001 (NCT04458922), representative PD-1 antibodies used in China, are undergoing clinical trials in adults and children. Detailed information on the clinical trials conducted in China related to PD-1/PD-L1 treatment is shown in online supplemental table 3.

Appropriate methods to identify pediatric patients who will benefit most from PD-1 therapy remain unclear. PD-L1 expression and the TMB are reported to be predictive biomarkers due to the strong association between the number of hypermutations and sensitivity to immunotherapy.^7^ An assessment of the effectiveness of a PD-1 antibody in patients with high-frequency microsatellite instability and biallelic mismatch repair deficiency^8^ (NCT02992964, NCT02332668, and NCT02359565) is understandable. The American Society of Clinical Oncology meeting in 2021 reported the preliminary study on the use of nivolumab in children with highly mutated tumors (NCT02992964).^9^ Results suggested that childhood tumors with a high tumor mutation load (TMB) and/or increased replication repair defects may be the susceptible to immune checkpoint inhibitor therapy. The overall best response was as follows: two patients (both with glioblastoma) achieved partial response (PR), and one patient with colorectal adenocarcinoma achieved pathological complete response (CR). The best response of the other five patients was stable disease, of which three patients were stable for more than 5 months. The median overall survival was not achieved.

Moreover, PD-1 and its ligands, PD-L1 and PD-L2, are overexpressed in Epstein Barr virus-positive lymphoproliferative disorders and are markers of aggressive behavior,^10^ which necessitates an assessment of the efficacy of PD-1 antibodies in these patients (NCT03258567). Furthermore, potential studies are set to begin for the purpose of identifying other pediatric patients with different pathologies, including melanoma (NCT04099251 and NCT03553836) or sarcoma (NCT03465592 and NCT03012620), who may experience positive effects of immunotherapy.^11^ Detailed information on the clinical trials of PD-1/PD-L1 monotherapy is provided in online supplemental table 4.

Seventy-four other drug targets are being tested in combination with PD-1/PD-L1 inhibitors. The three most common combinations are CTLA-4 monoclonal antibodies (11 trials), chemotherapy (nine trials) and therapies that target the vascular endothelial growth factor (VEGF) axis (seven trials) (figure 1A, B).

**Figure 1:**
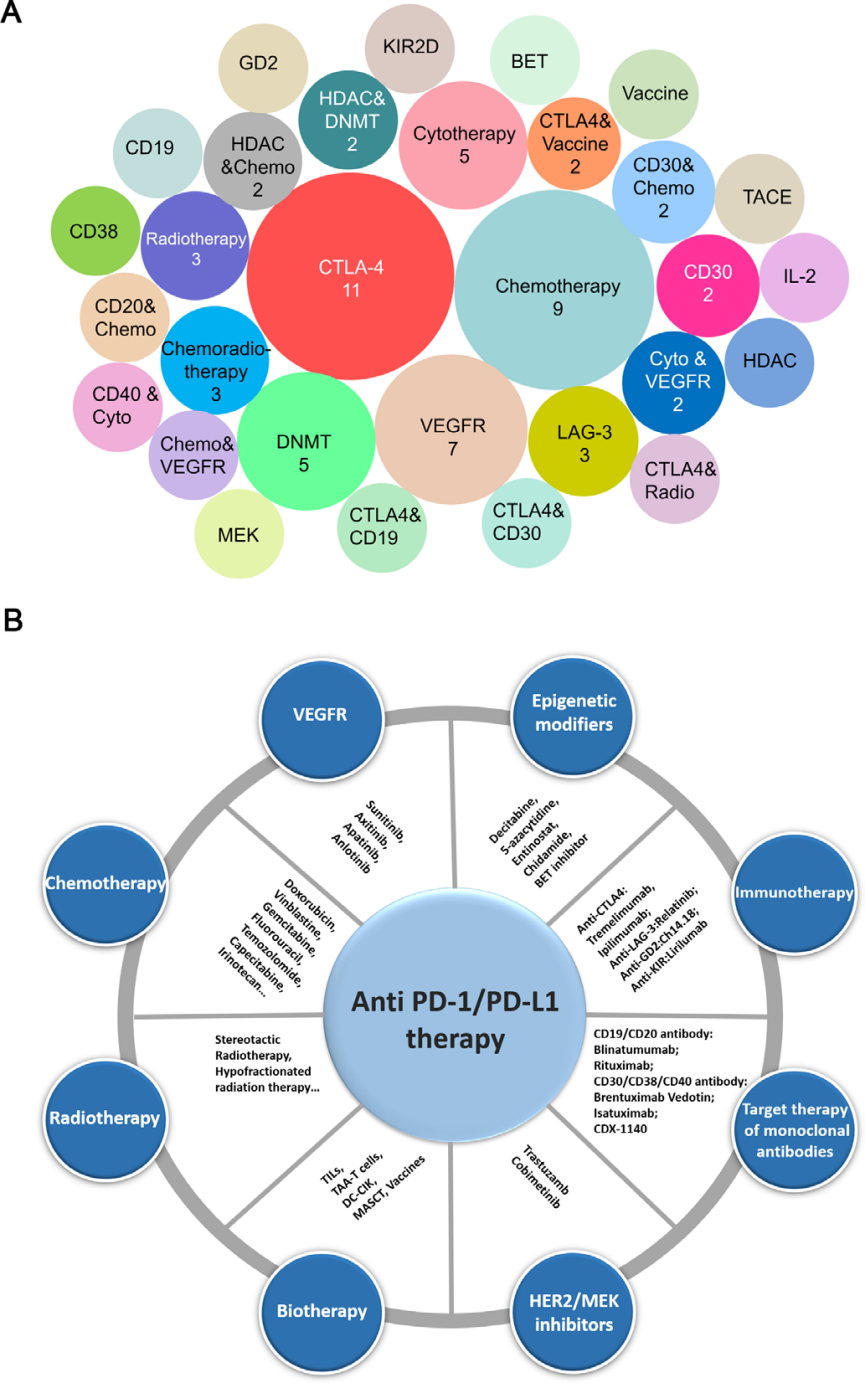
Combination trials/drugs with PD-1/PD-L1 inhibitors in pediatric patients. (A) Analysis of ongoing combination trials with PD-1/PD-L1 inhibitors conducted in the pediatric patients. The number of clinical trials for the therapy types is indicated in the labeled circles. The most popular combination is with CTLA-4 inhibitors, followed by chemotherapy, targeting either vascular endothelial growth factor or VEGFR, and then trial combination with DNMT. (B) Specific drug combinations with anti-PD-1/PD-L1 used to date. chemo, chemotherapy; cyto, cytotherapy; DC-CIK, dendritic cell-cytokine induced killer; DNMT, DNA methyltransferase; MASCT, multiple antigen-stimulating cellular therapy; PD-1, programmed cell death protein 1; PD-L1, cell death protein ligand 1; radio, radiotherapy; TAA-T, tumor-associated antigen-specific T cell; TIL, tumor-infiltrating lymphocyte; VEGFR, vascular endothelial growth factor.

The combination of PD-1/PD-L1 inhibitors and CTLA-4 inhibitors is considered a potential trend in immunotherapy.^12^ CTLA-4 inhibits the priming phase of T-cell activity by competing with CD28 for binding to CD28 ligands,^13^ while PD-1 regulates effector T-cell functions during the late phase of T-cell activity.^14^ Therefore, the combination of PD-1/PD-L1 and CTLA-4 blockade might be complementary to the mechanism. Cacciotti *et al*^15^ reported nine pediatric patients with recurrent/refractory CNS tumors who received ipilimumab and nivolumab in a retrospective study. Among them, three patients achieved PR; five had stable disease; and one had progressive disease.^15^ According to the case report by Pecora *et al*^16^, a 19-year-old man with stage IV epithelioid sarcoma, failed to respond to standard therapy and an EZH2 inhibitor. Then, the patient was observed for CR of the tumor after receiving one cycle of therapy with ipilimumab and nivolumab combination.^16^ At present, several clinical trials combining PD-1/PD-L1 inhibitors and anti-CTLA-4 therapy are under way (NCT03668119, NCT04323046, NCT04416568, NCT04465643, NCT04495010, NCT04500548, NCT02693535, NCT03837899, and NCT04480502) (online supplemental table 5). In addition, an ongoing study reported this year (2021-ASCO-TPS10055) has yet to release results on the combination of nivolumab and iplimumab in children with IN1-negative tumors.^17^

The efficacy of dual LAG-3 and PD-1 blockade in pediatric patients is unknown. To date, no specific clinical trials have been performed involving only children under the age of 18 for an assessment of this combination treatment. A phase I/IIa study—designed to assess the safety, tolerability and efficacy of an anti-LAG-3 monoclonal antibody (BMS-986016) alone and in combination with nivolumab in patients over the age of 12 with advanced solid tumors—is now under way (NCT01968109). Furthermore, two other clinical trials combining nivolumab and relatlimab are also currently recruiting patients with melanoma and chordoma (NCT03470922 and NCT03623854, respectively) (online supplemental table 5). LAG-3 is a promising immune checkpoint that shows a significant striking synergy with PD-1 in inhibiting T cell-mediated immune responses. Under the influence of PD-1 stimulation, LAG-3 expression on TILs is increased and functions to inhibit T-cell function and reduce chemokine secretion. Preclinical evidence also suggests that anti-LAG-3 is synergistically efficacious when combined with anti-PD-1.^18^ In addition, combination therapy with anti-LAG-3 and anti-PD-1 has provided remarkable clinical benefits to patients with melanoma.^19^ However, the efficacy of dual LAG-3 and PD-1 blockade in pediatric patients is still unknown. A preclinical study showed that the blockade of LAG-3, which is expressed in samples of human glioblastoma, cures glioblastoma tumors in mice when it is used alone or in combination with anti-PD-1.^20^

Epigenetic modifiers, including DNA methyltransferases and histone deacetylase inhibitors, may display potential synergistic efficacy with immunotherapy using anti-PD-L1/anti-PD-1 antibodies.^21^ DNA hypomethylation has been associated with elevated expression of PD-L1 in melanoma, indicating the potential therapeutic use of strategies targeting PD-L1 with DNA methylation inhibitors.^22^ Furthermore, preclinical studies showed the upregulation of PD-L1 expression by histone deacetylase inhibitors, thus increasing the sensitivity to anti-PD-1 therapy and inducing the secretion of T-cell chemokines in melanoma and lung adenocarcinoma.^23 24^ Notably, several ongoing clinical studies designed to measure the efficacy of PD-L1/PD-1 antibodies used in combination with epigenetic agents for pediatric patients with various cancers are presented in online supplemental table 6.

Preclinical studies have reported the potential efficacy of combining immune checkpoint therapy with antiangiogenic therapy, including multitargeted receptor tyrosine kinase inhibitors such as anlotinib, sunitinib, axitinib, and apatinib.^25–28^ One explanation for this synergistic effect is that immune checkpoint therapy might promote vessel normalization and inhibit angiogenesis to decrease immune checkpoint protein expression and reprogram the tumor microenvironment. Inspired by the significant survival advantage in preclinical studies, clinical studies in pediatric patients exploring the efficacy of anti-PD-1/PD-L1 in combination with anti-VEGF are under way (NCT03277924, NCT03595124, NCT03711279, NCT03946943, NCT04126993, NCT04447274, and NCT04503967). NCT04126993 is a phase II study measuring the effectiveness of SHR-1210 (camrelizumab) in combination with apatinib as a treatment for patients with unresectable sarcoma, in whom chemotherapy has proven to be ineffective. NCT03277924 is a phase I–II multicenter study assessing the efficacy of sunitinib plus nivolumab in adult and pediatric patients suffering from advanced soft tissue and bone sarcomas.

To our knowledge, early clinical studies of PD-1 antibodies used as monotherapy have demonstrated activity only in HL, hypermutant tumors, and some rare pediatric tumors but not the most common pediatric cancer types. The ACCELERATE and European Medicines Agency Pediatric Strategy Forum^29^ has recommended that immune-combination therapies in pediatric patients be explored on the basis of well-supported hypotheses, scientific data, and well-designed informative clinical trials. Furthermore, subjects concluded that synthetic immunotherapy, such as chimeric antigen receptor T cells and engineered antibody-based proteins, may be effective when combined with an immune checkpoint drug. In fact, strategies for combining PD-1/PD-L1 with other therapies have been developed in pediatric patients with cancer. Clinical trials are needed to reveal the potential and benefits of combination therapies in the pediatric population. PD-L1 expression alone is insufficient as a promising biomarker to identify children who might respond to immune checkpoint therapy. Therefore, studies aiming to explore and identify more molecular markers and targets to guide application of immune checkpoint therapy are needed. From preclinical studies, we found that the PD-1 and CTLA-4 antibodies may become the main combination and trend in pediatric cancer, as shown in figure 1. Although there are no pediatric tumor clinical trial results reported, PD-1/PD-L1 and CTLA-4 blockade might be complementary to the mechanism. In addition, the combination of antibodies targeting PD-1 with those targeting the immune checkpoint molecule CTLA-4 has shown particularly high response rates in adult patients with several malignancies, including metastatic melanoma. However, clinical trials with more high-level evidence are urgently needed to prove this hypothesis. In conclusion, this commentary provides a direction for designing clinical trials related to immunotherapy in pediatric patients with tumors.
