# Supplementary material for: Trends in clinical development of pediatric cancer for PD-1 and PD-L1 inhibitors: an analysis of ClinicalTrials.gov
Source: J Immunother Cancer. 2021 Sep 28;9(9):e002920. doi: 10.1136/jitc-2021-002920 (PMC8479973; doi:10.1136/jitc-2021-002920)
Supplement: Supplementary data [file jitc-2021-002920supp001.pdf]

Supplemental materials

Supplemental Figure 1. The landscape of ongoing PD-1/PD-L1 clinical trials in pediatric patients. A) Among the 98 interventional trials, 24 (24.5%) trials were related to PD-1/PD-L1 monoclonal antibodies alone, and 74 (75.5%) were related to PD-1/PD-L1 antibodies alongside other treatments(left). The percentage of ongoing PD-1 antibody clinical trials in pediatric patients according to the years (right). B) The proportion of clinical trials in each phase. C) Number of trials related to drugs anti-PD-1/PD-L1 in pediatric patients.

Que Y, *et al. J Immunother Cancer* 2021; 9:e002920. doi: 10.1136/jitc-2021-002920

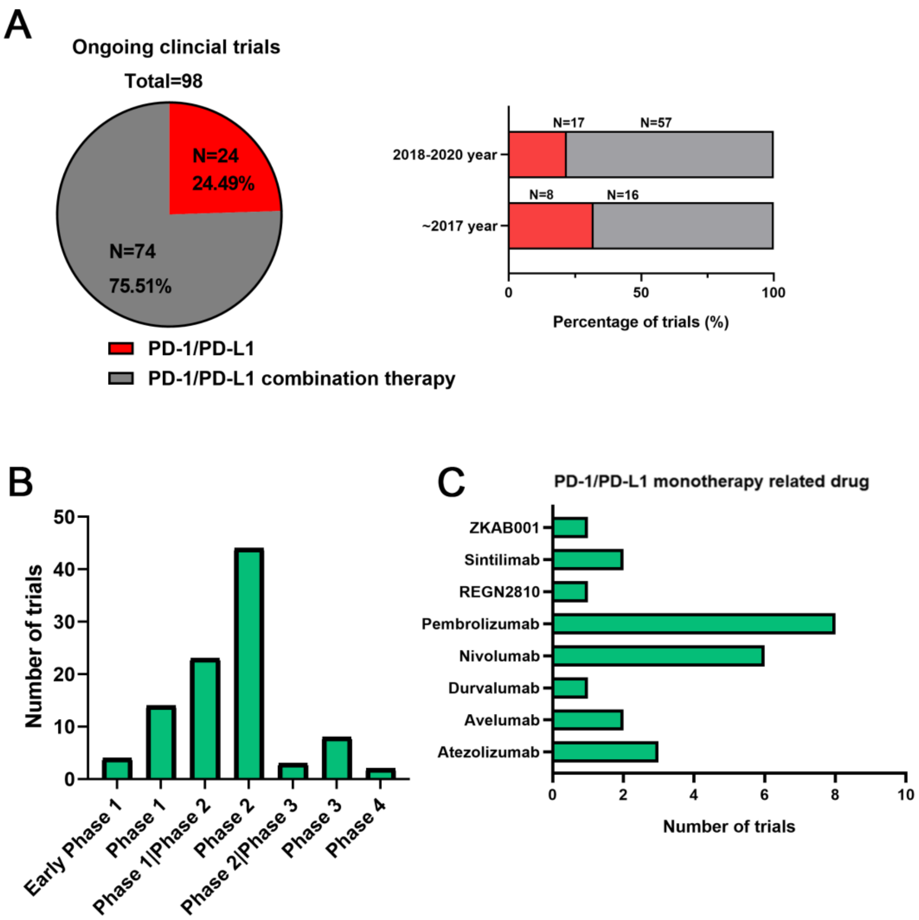
A
Ongoing clincial trials
Total=98
N=24
24.49%
N=74
75.51%
PD-1/PD-L1
PD-1/PD-L1 combination therapy
N=17
N=57
2018-2020 year
N=8
N=16
~2017 year
0
50
100
Percentage of trials (%)
B
Number of trials
50
40
30
20
10
0
Early Phase 1
Phase 1
Phase 1|Phase 2
Phase 2
Phase 2|Phase 3
Phase 3
Phase 4
C
PD-1/PD-L1 monotherapy related drug
ZKAB001
Sintilimab
REGN2810
Pembrolizumab
Nivolumab
Durvalumab
Avelumab
Atezolizumab
0
2
4
6
8
10
Number of trials

Supplemental Table 1. Summary of the main PD-1 and PD-L1 antibodies

**Therapeutic Drug**	**Manufacturer**	**Country**	**Target**
Nivolumab	Bristol-Myers Squibb	United States	PD-1
Pembrolizumab	Merck & Co	United States	PD-1
Toripalimab/ JS001	TopAlliance Biosciences	China	PD-1
Sintilimab/ IBI308	Innovent Biologics	China	PD-1
REGN2810	Regeneron& Sanofi	United States/France	PD-1
AMP-224	AstraZeneca	United Kingdom	PD-1
AMP-514	AstraZeneca	United Kingdom	PD-1
PDR-001	Novartis	Switzerland	PD-1
BCD-100	Biocad	Russia	PD-1
TSR-042	Tesaro	United States	PD-1
JNJ-63723273	Johnson & Johnson	United States	PD-1
PF--0681591	Pfizer Inc.	United States	PD-1
BI-754091	Boehringer Ingelheim	Germany	PD-1
Camrelizumab /SHR-1210	Jiangsu Hengrui Medicine Co.	China	PD-1
GB226	Genor Biopharma Co. Ltd	China	PD-1
GLS010	GLORIA Pharmaceuticals	China	PD-1
LZM009	Livzon Pharmaceutical Group Inc.	China	PD-1
HX008	Taizhou Hanzhong Biomedical Co.,	China	PD-1

BGB-A317	BeiGene	China	PD-1
Durvalumab	AstraZeneca	United States	PD-L1
Atezolizumab	Genentech	United States	PD-L1
Avelumab	Pfizer and Merck KGaA	United States	PD-L1
M7824	Merck & Co	United States	PD-L1
CX-072	CytomX Therapeutics	United States	PD-L1
FAZ-053	Novartis	Switzerland	PD-L1
LY-3300054	Eli Lilly and Company	United States	PD-L1
CA-170	Curis	United States	PD-L1
SHR-1316	Jiangsu Hengrui Medicine Co.	China	PD-L1
Envafolimab /KN035	Alphamab Oncology	China	PD-L1
ZKAB001	Lee's Pharmaceutial holdings limited	China	PD-L1
CX1001	CStone Pharmaceuticals	China	PD-L1
BAT1306	Bio-Thera Solutions, Ltd	China	PD-L1

7 **Supplemental Table 2. PD-1/PD-L1 checkpoint inhibitors in clinical trials in pediatric cancer**

Therapeutic Drug	Manufacturer	Target	Cancer type	NCT number	
Nivolumab	Bristol-Myers	PD-1	Hypermutant Cancers	NCT02992964	NCT04416568
	Squibb		EBV-Positive	NCT03258567	NCT04465643
			Non-HodgkinLymphomas	NCT03337919	NCT04495010
			Hodgkin Lymphoma	NCT03465592	NCT04500548
			Sarcoma	NCT03703050	NCT03808441
			Melanoma	NCT04099251	NCT02879695
			AML	NCT03825367	NCT01896999
			Gastric and Esophageal	NCT04503967	NCT04239040
			Cancer	NCT02914405	NCT04248569
			Neuroblastoma	NCT03595124	NCT02960230
			Kidney Cancer Called	NCT03628209	NCT02813135
			TFE/Translocation Renal Cell	NCT04410445	NCT03585465
			Carcinoma (tRCC)	NCT02419417	NCT03190174
			Recurrent, Resectable	NCT02927769	NCT03635983
			Osteosarcoma	NCT03712202	NCT02989636
			Unresectable Fibrolamellar	NCT03907488	NCT01968109
			Cancer	NCT03646123	NCT03470922
			Glioma	NCT03838042	NCT03623854
			Malignant Peripheral Nerve	NCT04380545	NCT03277924
			Sheath Tumor	NCT03668119	NCT03843294
			DIPG	NCT04323046	NCT04268888
			Metastatic Chordoma		NCT04267146

Que Y, *et al. J Immunother Cancer* 2021; 9:e002920. doi: 10.1136/jitc-2021-002920

Pembrolizumab	Merck & Co	PD-1	Lymphoma<br>High-Grade Gliomas, Diffuse Intrinsic Pontine Gliomas, Hypermutated Brain Tumors, Ependymoma or Medulloblastoma<br>Refractory Gray-Zone Lymphoma (GZL), Primary Central Nervous System Lymphoma (PCNSL), and Other Extranodal Diffuse Large B-cell Lymphomas<br>Melanoma<br>Merkel Cell Carcinoma<br>High-Risk Soft Tissue Sarcoma	NCT02332668<br>NCT02359565<br>NCT03012620<br>NCT03255018<br>NCT03532737<br>NCT03553836<br>NCT03783078<br>NCT04134559<br>NCT03092323<br>NCT02693535	NCT03605589<br>NCT04520711<br>NCT03407144<br>NCT03736330<br>NCT03445858<br>NCT03618550<br>NCT03719105<br>NCT04318717<br>NCT03769467<br>NCT03645928<br>NCT02621021
Atezolizumab	Genentech	PD-L1	Alveolar Soft Part Sarcoma<br>Clear Cell Sarcoma or Chondrosarcoma<br>DLBCL	NCT03141684<br>NCT03148418<br>NCT04458922<br>NCT04216953<br>NCT03422523<br>NCT00781612	
Avelumab	Pfizer and Merck KGaA	PD-L1	Peripheral T-cell Lymphoma<br>Classical Hodgkin Lymphoma	NCT03046953<br>NCT03617666	
Camrelizumab	Jiangsu Hengrui Medicine Co.	PD-1	Soft Tissue Sarcoma<br>Lymphoma	NCT03711279<br>NCT04126993	NCT04510610<br>NCT04351308

			UPS and ASPS	NCT04233294<br>NCT04514081<br>NCT03250962	NCT04225364<br>NCT04294511<br>NCT04447274<br>NCT03769181
Durvalumab	AstraZeneca	PD-L1	Pediatric Malignancies	NCT02793466<br>NCT03373760<br>NCT03837899	
Envafolimab	Alphamab	PD-L1	Undifferentiated Pleomorphic Sarcoma Or Myxofibrosarcoma	NCT04480502	
REGN2810	Regeneron&<br>Sanofi	PD-1	elapsed, Refractory Solid, or Central Nervous System (CNS) Tumors	NCT03690869	
Sintilimab	Innovent Biologics	PD-1	Advanced and Refractory Pediatric Malignant Tumors Neuroblastoma	NCT04400851<br>NCT04412408	
Toripalimab	TopAlliance Biosciences	PD-1	Undifferentiated Pleomorphic Sarcoma	NCT03946943	
ZKAB001	Lee's Pharmaceutial holdings limited	PD-L1	High-grade Osteosarcoma	NCT04359550	

Supplemental Table 3. Detailed information of clinical trials related to PD-1 /PD-L1 treatment conducted in China

Drug	Target	Study phase	Age	Cancer types	NCT	Status	Enrollment	Sponsor Country	Participating Country
Sintilimab	PD-1	Early Phase 1	12 Months to 12 Years	Recurrent Stage IV High Risk Neuroblastoma	NCT04412408	Not yet recruiting	10	China	NA
Sintilimab	PD-1	Phase 1	1 Year to 18 Years	Pediatric Cancer	NCT04400851	Recruiting	18	China	NA
ZKAB001	PD-L1	Phase 3	12 Years and older	High-grade Osteosarcoma	NCT04359550	Not yet recruiting	362	China	NA
Nivolumab+Anlotinib	PD-1+TKI	Phase 2	15 Years to 75 Years	Gastric Adenocarcinoma|Esophageal Squamous Cell Carcinoma	NCT04503967	Not yet recruiting	48	China	NA
Pembrolizumab+D-CIK+axitinib	PD-1+biological therapy+target therapy	Phase 2	Child, Adult, Older Adult	Renal Cancer Metastatic	NCT03736330	Recruiting	24	China	NA
Toripalimab+anlotinib	PD-1+TKI	Phase 2	16 Years and older	Soft Tissue Sarcomas|Undifferentiated Pleomorphic Sarcoma	NCT03946943	Not yet recruiting	25	China	NA

Camrelizumab+apatinib	PD-1+TKI	Phase 2	16 Years to 70 Years	Sarcoma	NCT03711279	Recruiting	289	China	NA
Camrelizumab+apatinib	PD-1+TKI	Phase 2	14 Years to 75 Years	Sarcoma	NCT04126993	Recruiting	80	China	NA
Carilizumab+apatinib	PD-1+TKI	Phase 2	16 Years and older	Sarcoma	NCT04447274	Not yet recruiting	20	China	NA
Camrelizumab+Neoadjuvant Chemotherapy	PD-1+Chemotherapy	Phase 2	14 Years to 65 Years	Osteosarcoma	NCT04294511	Recruiting	75	China	NA
Camrelizumab+Neoadjuvant Chemotherapy	PD-1+chemotherapy	Phase 2	17 Years to 70 Years	Esophageal Squamous Cell Carcinoma	NCT04225364	Recruiting	50	China	NA
Camrelizumab+MAPI chemotherapy+Apatinib	PD-1+chemotherapy+TKI	Phase 2	12 Years and older	Osteosarcoma	NCT04351308	Recruiting	60	China	NA
Camrelizumab+Decitabine	PD-1+DNA Methyltransferase inhibitor	Phase 2|Phase 3	12 Years to 75 Years	Hodgkin Lymphoma	NCT04510610	Recruiting	100	China	NA
Camrelizumab+Decitabine	PD-1+DNA Methyltransferase inhibitor	Phase 2	12 Years to 75 Years	Hodgkin Lymphoma	NCT03250962	Recruiting	280	China	NA
Camrelizumab+Chidamide +Decitabine	PD-1+HDAC inhibitor+ DNA Methyltransfe	Phase 2	12 Years to 75 Years	Hodgkin Lymphoma|Anti-PD-1 Antibody Resistant	NCT04514081	Recruiting	200	China	NA

	rase inhibitor								
Camrelizumab+Chidamide +Decitabine	PD-1+HDAC inhibitor+ DNA Methyltransferase inhibitor	Phase 2	12 Years to 75 Years	Hodgkin Lymphoma	NCT04233294	Recruiting	100	China	NA

Supplemental Table 4. Detailed information of clinical trials related to PD-1 /PD-L1 treatment in pediatric patients

**Drug**	**Target**	**Study phase**	**Age**	**Cancer types**	**NCT**	**Status**	**Enrollment**	**Sponsor Country**	**Participating Country**
Nivolumab	PD-1	Phase 1|Phase 2	12 Months to 18 Years	Refractory or Recurrent Hypermutated Malignancies|Biallelic Mismatch Repair Deficiency (bMMRD) Positive Patients	NCT02992964	Recruiting	50	Canada	Australia, United States, France, Israel
Nivolumab	PD-1	Phase 2	12 Years and older	Epstein-Barr Virus Infections|Lymphoma|Lymphoproliferative Disorder|Disorders, Lymphoproliferative	NCT03258567	Recruiting	80	United States	NA
Nivolumab	PD-1	Phase 2	16 Years and older	Hodgkin Lymphoma	NCT03337919	Recruiting	120	United Kingdom	NA
Nivolumab	PD-1	Phase 1|Phase 2	12 Months to 40 Years	Sarcoma|Solid Tumor, Adult|Solid Tumor, Childhood	NCT03465592	Recruiting	39	United States	NA
Nivolumab	PD-1	Phase 2	6 Months and older	Relapsing/Refractory ALK+ Anaplastic Large Cell Lymphoma	NCT03703050	Recruiting	38	France	Denmark, Netherlands
Nivolumab	PD-1	Phase 3	12 Years and older	Melanoma	NCT04099251	Recruiting	1000	United States	Australia, Austria,

									Belgium, Canada, et al.
Pembrolizumab	PD-1	Phase 1|Phase 2	6 Months to 17 Years	Melanoma|Lymphoma|Solid Tumor|Classical Hodgkin Lymphoma|Microsatellite-instability-high Solid Tumor	NCT02332668	Recruiting	310	United States	Australia, Brazil, France, Germany, et al.
Pembrolizumab	PD-1	Phase 1	1 Year to 29 Years	Constitutional Mismatch Repair Deficiency Syndrome|Lynch Syndrome|Malignant Glioma|Recurrent Brain Neoplasm|Recurrent Childhood Ependymoma|Recurrent Diffuse Intrinsic Pontine Glioma|Recurrent Medulloblastoma|Refractory Brain Neoplasm|Refractory Diffuse Intrinsic Pontine Glioma|Refractory Ependymoma|Refractory Medulloblastoma	NCT02359565	Recruiting	110	United States	NA
Pembrolizumab	PD-1	Phase 2	15 Years and older	Sarcoma|Ovarian Neoplasm|Central Nervous System Neoplasm|Thyroid Neoplasm|Carcinoma, Neuroendocrine|Neoplasms, Germ Cell and Embryonal|NK/T-cell Lymphoma	NCT03012620	Recruiting	350	France	NA
Pembrolizumab	PD-1	Phase 2	14 Years and older	Non-Hodgkin Lymphoma|Lymphoma|Diffuse Large B-Cell Lymphoma|Gray Zone Lymphoma|Primary Central Nervious System Lymphoma	NCT03255018	Recruiting	52	United States	NA
Pembrolizumab	PD-1	Phase 2	16 Years and older	Locally Advanced Head and Neck Cancer	NCT03532737	Recruiting	50	Kuwait	NA
Pembrolizumab	PD-1	Phase 3	12 Years and older	Melanoma	NCT03553836	Recruiting	954	United States	Australia, Belgium, Brazil, Canada, et al.

Que Y, *et al. J Immunother Cancer* 2021; 9:e002920. doi: 10.1136/jitc-2021-002920

Pembrolizumab	PD-1	Phase 3	12 Years and older	Merkel Cell Carcinoma	NCT03783078	Recruiting	50	United States	Australia, Canada, France, Italy, et al.
Pembrolizumab	PD-1	Phase 2	up to 30 Years	Hepatocellular Carcinoma, Childhood|Hepatocellular Carcinoma|Liver Cancer|Liver Cancer Pediatric	NCT04134559	Recruiting	18	United States	NA
REGN2810	PD-1	Phase 1|Phase 2	up to 25 Years	Relapsed Solid Tumor|Refractory Solid Tumor|Relapsed Central Nervous System Tumor|Refractory Central Nervous System Tumor|Diffuse Intrinsic Pontine Glioma|High Grade Glioma	NCT03690869	Recruiting	130	United States	NA
Sintilimab	PD-1	Phase 1	1 Year to 18 Years	Pediatric Cancer	NCT04400851	Recruiting	18	China	NA
Sintilimab	PD-1	Early Phase 1	12 Months to 12 Years	Recurrent Stage IV High Risk Neuroblastoma	NCT04412408	Not yet recruiting	10	China	NA
Durvalumab	PD-L1	Phase 1	1 Year to 17 Years	Solid Tumor|Lymphoma|Central Nervous System Tumors	NCT02793466	Recruiting	36	United States	NA
Avelumab	PD-L1	Phase 2	16 Years and older	T-Cell Lymphoma Relapsed|T-Cell Lymphoma Refractory	NCT03046953	Recruiting	35	United Kingdom	NA
Avelumab	PD-L1	Phase 2	16 Years to 60 Years	Hodgkin Lymphoma	NCT03617666	Recruiting	47	United Kingdom	NA
Atezolizumab	PD-L1	Phase 2	2 Years and older	Metastatic Alveolar Soft Part Sarcoma|Unresectable Alveolar Soft Part Sarcoma	NCT03141684	Recruiting	46	United States	NA
Atezolizumab	PD-L1	Phase 3	Child,	Cancer	NCT03148418	Recruiting	380	Switzerland	United States,

			Adult, Older Adult						Argentina, Australia, Austria, Belgium, et al.
Atezolizumab	PD-L1	Phase 2	2 Years and older	Chondrosarcoma NCI Grade 2|Chondrosarcoma NCI Grade 3|Clear Cell Sarcoma of Soft Tissue|Dedifferentiated Chondrosarcoma|Primary Central Chondrosarcoma	NCT04458922	Not yet recruiting	60	United States	NA
ZKAB001	PD-L1	Phase 3	12 Years and older	High-grade Osteosarcoma	NCT04359550	Not yet recruiting	362	China	NA

Supplemental Table 5. Detailed information of clinical trials related to combination of PD-1 /PD-L1 inhibitors with CTLA-4 or LAG-3 inhibitors in pediatric patients

**Drug**	**Target**	**Study phase**	**Age**	**Cancer types**	**NCT**	**Status**	**Enrollment**	**Sponsor Country**	**Participating Country**
Nivolumab+Ipilimumab	PD-1+CTLA4	Phase 2	12 Years and older	Pan Tumor	NCT03668119	Recruiting	183	United States	Argentina, Australia, Canada, Chile,

									et al.
Nivolumab+Ipilimumab	PD-1+CTLA4	Phase 1	6 Months to 22 Years	Glioblastoma	NCT04323046	Not yet recruiting	45	United States	Australia, Israel, Switzerland
Nivolumab+Ipilimumab	PD-1+CTLA4	Phase 2	6 Months to 30 Years	Malignant Rhabdoid Tumor	NCT04416568	Recruiting	45	United States	NA
Nivolumab+Ipilimumab	PD-1+CTLA4	Phase 1	12 Years to 100 Years	Nerve Sheath Tumors	NCT04465643	Not yet recruiting	18	United States	NA
Nivolumab+Ipilimumab	PD-1+CTLA4	Phase 2	12 Years and older	Melanoma	NCT04495010	Not yet recruiting	657	United States	Australia, Belgium, Brazil, Denmark, et al.
Nivolumab+Ipilimumab	PD-1+CTLA4	Phase 1	12 Months to 25 Years	Hematopoietic Refractory Lymphoma|Refractory Malignant Solid Neoplasm|Refractory Neuroblastoma|Refractory Primary Central Nervous System NeoplasM	NCT04500548	Not yet recruiting	40	United States	Canada
Nivolumab+Ipilimumab	PD-1+CTLA4	Phase 2	12 Years and older	Lymphoma, Non-Hodgkin|Multiple Myeloma|Advanced	NCT02693535	Recruiting	3279	United States	NA

				Solid Tumors					
Durvalumab+Tremelimumab	PD-L1+CTLA4	Phase 2	Child, Adult, Older Adult	Stage IV Squamous Cell Lung Carcinoma	NCT03373760	Active, not recruiting	132	United States	NA
Durvalumab+Tremelimumab	PD-L1+CTLA4	Phase 1|Phase 2	up to 18 Years	Pediatric Cancer|Solid Tumor Pediatric|Hematological Malignancies	NCT03837899	Recruiting	158	United Kingdom	United States, France, Germany, Italy, Netherland, et al.
Envafolimab+Ipilimumab	PD-L1+CTLA4	Phase 2	12 Years and older	Undifferentiated Pleomorphic Sarcoma|Myxofibrosarcoma	NCT04480502	Not yet recruiting	160	United States	NA
Nivolumab+Relatlimab	PD-1+LAG-3	Phase 1|Phase 2	12 Years and older	Neoplasms by Site	NCT01968109	Recruiting	1500	United States,	Australia, Austria, Canada, Denmark, et al.
Nivolumab+Relatlimab	PD-1+LAG-3	Phase 2|Phase 3	12 Years and older	Melanoma	NCT03470922	Recruiting	700	United States	Argentina, Australia, Austria, Belgium, et al.
Nivolumab+Relatlimab	PD-1+LAG-3	Phase 2	12 Years and older	Chordoma|Locally Advanced Chordoma|Metastatic	NCT03623854	Recruiting	20	United States	NA

				Chordoma|Unresectable Chordoma					

Supplemental Table 6. Detailed information of clinical trials related to combination of PD-1 /PD-L1 inhibitors with epigenetics modifiers in pediatric patients

Drug	Target	Study phase	Age	Cancer types	NCT	Status	Enrollment	Sponsor Country	Participating Country
Camrelizumab+Decitabine	PD-1+DNA Methyltransferase inhibitor	Phase 2	12 Years to 75 Years	Hodgkin Lymphoma	NCT03250962	Recruiting	280	China	NA
Nivolumab+Azacitidine	PD-1+DNA methyltransferase inhibitor	Phase 1|Phase 2	up to 39 Years	Osteosarcoma|Osteosarcoma in Children|Osteosarcoma Recurrent|Sarcoma	NCT03628209	Recruiting	51	United States	NA
Nivolumab+5-azacytidine	PD-1+DNA methyltransferase inhibitor	Phase 1|Phase 2	1 Year to 30 Years	AML, Childhood	NCT03825367	Recruiting	26	United States	NA
Camrelizumab+Decitabine	PD-1+DNA	Phase	12	Hodgkin Lymphoma	NCT04510610	Recruiting	100	China	NA

	methyltransferase inhibitor	2|Phase 3	Years to 75 Years						
Nivolumab+Entinostat	PD-1+HDAC inhibitor	Phase 1|Phase 2	6 Years to 21 Years	CNS Tumor|Solid Tumor	NCT03838042	Recruiting	128	Germany	Australia, France, Austria, Netherlands, Sweden
Camrelizumab+Chidamide +Decitabine	PD-1+HDAC inhibitor+DNA methyltransferase inhibitor	Phase 2	12 Years to 75 Years	Hodgkin Lymphoma	NCT04233294	Recruiting	100	China	NA
Camrelizumab+Chidamide +Decitabine	PD-1+HDAC inhibitor+DNA methyltransferase inhibitor	Phase 2	12 Years to 75 Years	Hodgkin Lymphoma|Anti-PD-1 Antibody Resistant	NCT04514081	Recruiting	200	China	NA
